# Glycyrrhetinic Acid Induces Apoptosis in Leukemic HL60 Cells Through Upregulating of CD95/ CD178

**Published:** 2014

**Authors:** Sara Pirzadeh, Shohreh Fakhari, Ali Jalili, Sako Mirzai, Bayazeed Ghaderi, Venous Haghshenas

**Affiliations:** 1 *Department of Biochemistry, Research & Development, Islamic Azad University, Sanandaj, Iran.*; 2 *Kurdistan Cellular & Molecular Research Center, Kurdistan University of Medical Sciences, Sanandaj, Iran.*

**Keywords:** Acute leukemia, glycyrrhetinic acid, cytotoxicity, apoptosis

## Abstract

Acute leukemia is characterized by the accumulation of neoplastic cells in the bone marrow and peripheral blood. Currently, chemotherapy and differentiating agents have been used for the treatment of leukemia. Recently, plant extracts, either alone or in combination with chemo agents, have been proposed to be used for the treatment of cancers. The aim of the present research was to study the cytotoxicity and apoptosis effects of an active licorice-derived compound, glycyrrhetinic acid (GA), on human leukemic HL60 cells. HL60 cells were cultured in RPMI1640 containing 10% fetal bovine serum. Cells were treated with different doses of GA and their viability and proliferation were detected by dye exclusion and 3-bis-(2-methoxy-4-nitro-5-sulfophenyl)-2H-tetrazolium-5-carboxanilide (XTT) assays. Apoptosis induction and expression of CD95 and CD178 were analyzed by flow cytometry. We observed that GA decreases cell viability and suppresses cells proliferation in a dose- dependent manner. In addition, our flow cytometry data show that GA not only induces apoptosis in HL60 cells, but also upregulates both CD95 and CD178 expression on the cell surface of these cells in a dose-dependent manner. The combination of GA with cytotoxic drugs and differentiation agents requires further investigation.

Acute leukemia is characterized by accumula-tion of leukemic blast cells in the bone marrow and peripheral blood. Although chemothe-rapy and clinical use of differentiating agents are therapeutic procedures for the treatment of leukemia, these conventional treatments have many side effects and deficiencies including drug-resistance (1, 2).

Many new findings suggest that most of the cell growth regulating mechanisms are damaged in the process of cancer initiation and consequently cell growth gets out of the body’s control. Apoptosis or programmed cell death is one of the fundamental mechanisms that regulates cell growth and death (3). Basically, apoptosis is activated by CD95 which binds to its ligand, CD178 on the activating cell which is often a lymphocyte (4). Interaction of CD95 and CD178 leads in activation of death domains which subsequently results in activation of caspase and cell death (4, 5). On the other hand, previous studies demonstrated that many cancers particularly leukemic cells became resistant to apoptosis (3, 6).

Many recent studies have focused on the potential application of natural products for treatment of cancers including leukemia. Recent findings have suggested that medicinal plants are valuable resources for production of pharmaceutical products and chemo drugs (7, 8). Licorice is a traditional herbal medicine originated from the licorice root, and it remains one of the most commonly prescribed herbs in Chinese and Persian medicine (9, 10). Glycyrrhetinic acid β (GA), the hydrolyzed metabolite of glycyrrhizin (GZ), exhibits many anti- inflammatory and anti- viral effects. Moreover, previous studies revealed that GA and GZ showed toxic effects against many cancer cells including liver, prostate and colon (-). However, its underlying mechanism still needs to be explored.

The aim of this study was to evaluate the cytotoxic effects of GA against the growth and proliferation of HL60 cells as a promyelocytic leukemia cell line and investigate its effects on CD95 and CD178 expression as activators of apoptosis.

## Material and Methods

**Cell culture**

The HL60 cell line (human promyelocytic leukemia cell line) was purchased from Pasteur Institute of Iran (Tehran, Iran) and was cultured in RPMI 1640 (Gibco, Manchester, UK) containing 10% FBS, at 37°C in a humid incubator with 5% CO2.

**Cell viability**

To examine the effect of GA on cell viability of HL60 cells, 2x 10^5^ cells were cultured in the presence of different concentrations of GA (Sigma, Munich, Germany) in RPMI 1640 containing 10% FBS for either 24 or 48 hours. Then, cells viability was determined by dye exclusion assay and cells were counted using hemocytometer slides. The IC50 value, the concentration of GA where 50% of cells are viable, was determined.

**XTT assay**

The assay detects the reductiom of XTT (2, 3-bis-(2- methoxy- 4- nitro- 5- sulfophenyl)- 2H-tetrazolium-5- carboxanilide) to formazon product which reflects the normal function of mitochondria. Thus, XTT assay (CCK-8, Dojindo,Tokyo, Japan) was employed to assess the toxic effects of GA on HL60 cells. 4x 10^3^ cells were added to each well of 96 well plates (in duplicate) with or without different concentrations of GA in RPMI 1640 containing 10% FBS. Cells were incubated at 37 ° C in a humidified incubator with 5% CO2 for either 24 h or 48 h, and then 10 µL of CCK-8 was added to each well and incubated in incubator for 4 h. The optical density (OD) was measured at 450 nm using a microplate reader (Stat Fax, Palm City, FL).

The absorbance of untreated cells was considered as 100%. Percentage growth inhibition was calculated as previously reported. Percentage of inhibition= 100- [(test OD/ untreated OD)x 100] (14).

**Apoptosis assay**

In order to examine whether GA treatment could result in apoptosis induction, cells were treated with various concentrations of GA and apoptosis was examined using Annexin-V/PI kit (ebioscience, San Diego, CA). HL60 cells were treated with different concentrations of GA for 24 h and stained for Annexin V and PI according to the manufacturer's instructions. Briefly, cells were harvested, washed and incubated with 5 µL of Annexin V and 10 µL PI for 15 min in dark place. Next, after washing with 300 µL binding buffer, the cells were gently resuspended in 300 µL of binding buffer, kept on ice and subjected to FCAS analysis (FACS Calibur, Beckman Dickinson, San Jose, CA) within an hour. Flow cytometry data were analyzed by FCS Express software (De Novo Software, Los Angeles, CA).

**Flow cytometry analysis of CD95 and CD178 expression**

To examine the possible effects of GA on expression of CD95 and CD178, we incubated the cells with different concentrations of GA for 24 h. The levels of CD95 and CD178 expression on the surface of HL60 cells were determined by flow cytometry. 5x 10^5^ cells were counted and washed three times with FCM buffer (PBS containing 0.1% BSA) and stained with either 10 µL of PE- conjugated control antibody or mouse anti- human CD95- PE or mouse anti- human CD178- PE (all from Biolegend, San Diego, CA) for 45 min at 4 Cº as previously reported (15). Cells were then washed three times with FCM buffer, fixed in 1% paraformaldehyde, and subjected to FCAS analysis ((FACS Calibur, Beckman Dickinson, San Jose, CA). Flow cytometry data were analyzed by FCS Express software (De Novo Software, Los Angeles, CA).

**Statistical analyzes**

Arithmetic means and standard deviations were calculated and statistical significance was defined as P ≤ 0.05 using Student’s *t-*test.

## Results

**GA inhibits cell viability and proliferation**

**Fig. 1:**
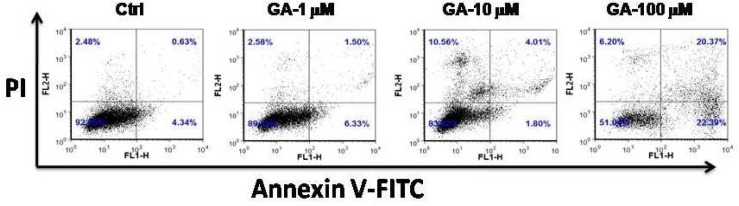
GA reduces cell viability and proliferation in a dose dependent manner. (A) Effect of increasing concentrations of GA on HL60 cells viability after either 24 h or 48 h. (B) Effect of GA on cell proliferation. Data are represented as Mean± SEM of three independent experiments. P< 0.05

**Fig. 2:**
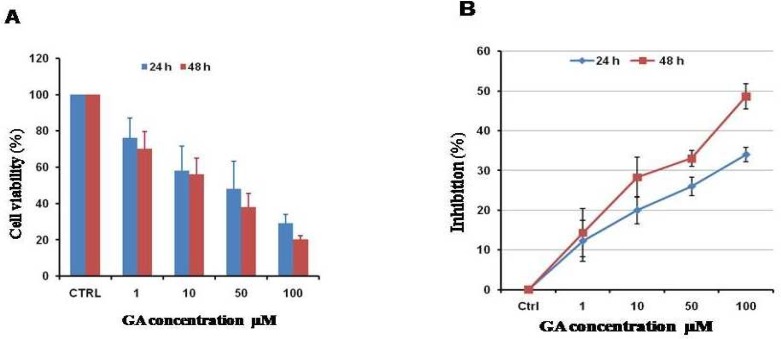
Effects of GA on cell growth of HL60 cells. Cells were incubated with increasing concentrations of GA for 24 h. Representative microscopic photos are shown

First, we examined the effects of GA on the viability of HL60 cells and observed that this licorice- derived compound is capable to decrease cell viability in a dose- dependent manner (Figure 1A). Moreover, the effect of GA on cell proliferation was evaluated using XTT assay. Our data show that GA inhibits cell proliferation in a dose- dependent manner (Figure 1B) confirming the cell counting data. Moreover, we observed that upon 48 h treatment with GA, cells viability and proliferation were slightly more reduced in comparison to 24 h treatment. The IC50 of GA on HL60 cells was 38±11 and 25±9 μM when the cells were treated for 24 and 48 h, respectively. Figure 2 clearly shows that numbers of cells were decreased in the presence of 50 and 100 µM of GA.

**Effect of GA on apoptosis induction in HL60 cells**

**Fig. 3:**
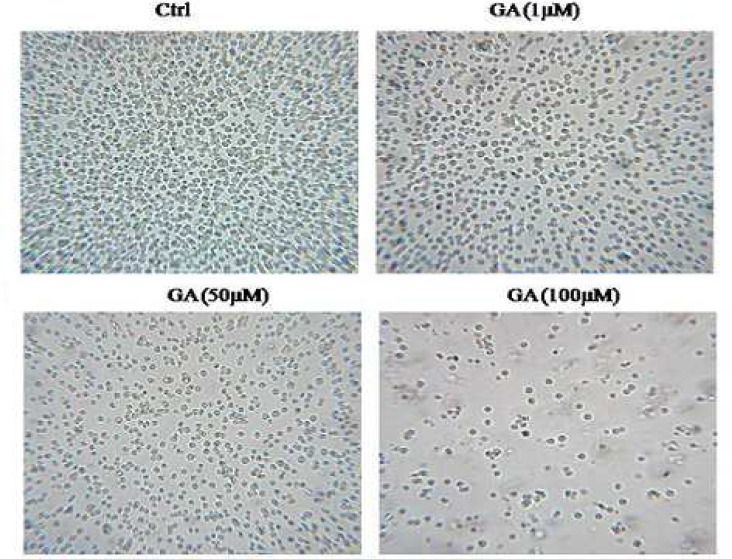
GA induces apoptosis in HL60 cells. Cells were incubated with increasing concentration of GA for 24 h. Apoptosis induction was evaluated by flow cytometry within 1h. Representative data from three independent experiments are shown

Having shown that GA reduces cell viability, we sought to examine the effect of GA on inducing apoptosis in these leukemic cancer cells. To do this, the cells were treated with various concen-trations of GA and apoptosis was measured by detection of Annexin V and PI using flow cytometry. As demonstrated in figure 3, GA induced apoptosis in HL60 cells in a dose- dependent manner.

**GA upregulates expression of both CD95 and CD178 on the cell surface of HL60 cells**

**Fig. 4:**
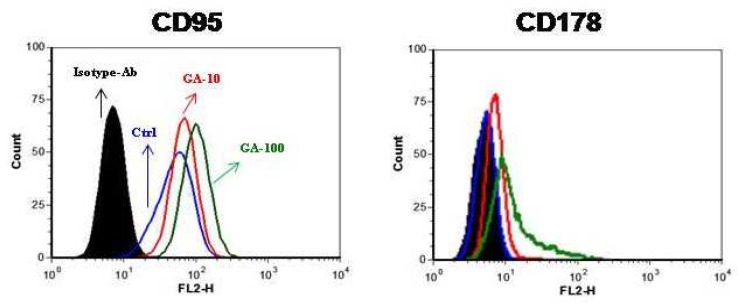
GA upregulates CD95 and CD178 on the cell surface of HL60 cells. Cells were incubated with increasing concentration of GA for 24 h. CD95 and CD178 expression was analyzed by flow cytometry. Black-filled and blue histograms represent isotype control antibody and control cells, respectively. Red and green histograms show 10 and 100 µM of GA- treated cells, respectively. Representative data from three independent experiments are shown

As CD95 and its ligand CD178, play a crucial role in apoptosis, we examined the effect of GA on the expression of CD95 on cell surface of HL60 cells and found that CD95 is expressed on the cell surface of HL60 cells and GA upregulates it in a dose-dependent manner (Figure 4 A). Interestingly, we observed that CD178 is not expressed on the cell surface of HL60 cells, but its expression enhanced when the cells were treated with GA (Figure 4B).

## Discussion

Currently, various therapeutic methods are being used to destroy malignant leukemic clones. Chemotherapy is the most common treatment for eradicating leukemia and inducing complete remission. But due to the severe side effects of chemotherapy, alternative and less toxic pharmaceutical agents have been considered. Recently, increasing attention has been focused on the application of natural products in cancer therapy. Among them, GA has been shown to have cytotoxic activities against many cancerous cells (9). Herein, we treated HL60 cells with GA and observed that this licorice- derived compound induces apoptosis in HL60 and upregulates CD95 and CD178 on these cells as well.

Xiu-ying Xiao et al. analyzed the effects of seven licorice- derived components including GA and GZ on gastric cancer cells such as MKN-28, AGS, and MKN-45 cells. They observed that licochalcone A was the most cytotoxic component of the extract and showed the highest effect on apoptosis induction and cell growth inhibition (16). In contrast to our data, this previous report showed that GA was only toxic to gastric cell lines when treated with a high dose of GA (16). Similarly, another study has reported that GA is not capable to induce apoptosis in rat hepatocytes (17). These discrepancies could be explained by differences in cell lines and experimental conditions. The current study demonstrates that a much lower dose of GA inhibits HL60 cells growth and proliferation. Although, GA displays a cytotoxic activity against HL60 in a dose- dependent manner, it did not show significant cytotoxic effects against HL60 cells when the cells were treated for a longer time (48 h), indicating that 24 h might be good enough for GA to exhibit its activity on this particular leukemic cells.

Mounting evidence indicates that many chemo drugs induce apoptosis in leukemic cells (2). However, leukemic cells become resistant when they express multi- drug resistant molecules such as P-glycoprotein (18) implying that alternative pharmaceutical agents are necessary for eradicating leukemic cells. In the current study, we have found that GA induces apoptosis in HL60 in a dose- dependent manner, indicating that GA could be a suitable candidate for combination therapy. Supporting this notion is a previous study which demonstrated that combination of GA with doxorubicin revealed a synergistic toxic effect on human cervical cancer SiHa cells (19).

CD95/ CD178 interaction is involved in signaling transduction pathway of apoptosis and its role in chemotherapy- induced apoptosis of a number of tumors have already been investigated (20, 21). CD95 is also a tumor necrosis factor receptor superfamily member 6 which is a glycosylated cell surface molecule localized on the cell surface and cytoplasm of many cells and its upregulation during induction of apoptosis has been displayed in many tumors cells (22). Ligation of CD95 to CD178 induces receptor oligomerization and formation of death receptor signaling complex resulting in the activation of a series of caspase enzymes which in turn lead into cell death (4). Our study shows that GA upregulates CD95 expression on the cell surface of HL60 and that GA-mediated CD95 upregulation may contribute, at least partially to the cell death of CD95- expressing cells. Noteworthy, it has been shown that CD178-expressing tumor cells kill the immune cells which have a high level of CD95 on their surface (23). Accordingly, Salih et al. have recently shown that retinoic acid and vitamin E reduce CD178 in carcinoma cells and those treated cells show a lower ability to kill CD95- sensitive cells (24). On the other hand, a recent study demonstrated that licochalcone A, another active compound of licorice, induces apoptosis in oral cancer cells via CD178- mediated death receptor pathway (25). Having shown that GA upregulates expression of both CD95 and CD178 on the cell membrane of HL60 cells, we postulate that the interaction of elevated CD95 and CD178 could lead to the induction of apoptosis in GA- treated HL60 cells.

In conclusion, we herein demonstrated that GA exhibits cytotoxic activities against HL60 cells by induction of apoptosis and that GA- induced apoptosis is at least partially initiated by interaction of CD95 and CD178 signaling pathway.
